# Study of phosphorylation events for cancer diagnoses and treatment

**DOI:** 10.1186/s40169-015-0059-0

**Published:** 2015-05-24

**Authors:** Elena López Villar, Luis Madero, Juan A López-Pascual, William C Cho

**Affiliations:** Oncohematology of Children Department, Hospital Universitario Infantil Niño Jesús, Av. Menéndez Pelayo 65, Madrid, Spain; Oncohematology Department, Hospital QUIRÓN Madrid, Madrid, Spain; Department of Clinical Oncology, Queen Elizabeth Hospital, Hong Kong, China

**Keywords:** Diagnosis, Oncology, Proteomics, Treatment

## Abstract

The activation of signaling cascades in response to extracellular and intracellular stimuli to control cell growth, proliferation and survival, is orchestrated by protein kinases *via* phosphorylation. A critical issue is the study of the mechanisms of cancer cells for the development of more effective drugs. With the application of the new proteomic technologies, together with the advancement in the sequencing of the human proteome, patients will therefore be benefited by the discovery of novel therapeutic and/or diagnostic protein targets. Furthermore, the advances in proteomic approaches and the Human Proteome Organization (HUPO) have opened a new door which is helpful in the identification of patients at risk and towards improving current therapies. Modification of the signaling-networks *via* mutations or abnormal protein expression underlies the cause or consequence of many diseases including cancer. Resulting data is used to reveal connections between genes proteins and compounds and the related molecular pathways for underlining disease states. As a delegate of HUPO, for human proteome on children assays and studies, we, at Hospital Universitario Niño Jesús, are seeking to support the human proteome in this context. Clinical goals have to be clearly established and proteomics experts have to set up the appropriate proteomic strategy, which coupled to bioinformatics will make it possible to achieve new therapies for patients with poor prognosis. We envision to combine our up-coming data to the HUPO organization in order to support international efforts to advance the cure of cancer disease.

## Background

Proteomics is a powerful tool in biomarker discovery and mechanism understanding. Proteomics is the next generation following genomics. Using proteomics, researchers can efficiently perform large-scale screening to achieve valuable information [1].

The Human Proteome Organization (HUPO) main goal is to serve the Public Health Service in an international manner *via* collaborators from the best expertise and well-known excellence academics. In this context, we would like to point out the important contribution from Spanish Proteomic Society (SeProt), European Proteomic Society (EUPA) and HUPO to obtain the sequence of the human proteome. We aim to contribute with our future data from Hospital Niño Jesus using body fluids (such as bone marrow, peripheral blood) from children suffering cancer, and by collaboration with several international hospitals and research centers of prestige for this important labor, which will benefit children with poor prognoses as at present there is no correct therapy for all types of pediatric cancers [2–5].

As different phosphosites in a protein trigger either protein activation or inactivation; phosphosites can be used for quantification. The high number which can be identified as altered phosphoproteins in a clinical study, implies that many can also be reported with key roles in tumor progression and/or drug resistance. Indeed, when obtaining phosphoproteomics data in clinical research, vast knowledge of drug resistance appear, and thus, new insights are offered for future drug candidates. Several articles show that proteomic analysis is a powerful tool for profiling the phosphorylation patterns and may help to better understand drug resistance [6–11]. We aim to show some proteomic and bioinformatics tools which are useful for clinical research and which contribute towards accurate diagnoses and improve therapies in order to benefit the patients.

## Findings

### Phosphoproteomics for deciphering drug resistance and new therapies related to phosphorylation pathways in cancer

Our aim is to outline as basic ideas and tools of proteomics, how evolution is aiming to reach clinical advances. Amino acids site-specific phosphorylation assignments on thousands of proteins in a single experiment are possible. The combination of different proteomic-MS strategies is already being carried out to characterize signaling pathways that govern oncogenesis and also to unravel targets of kinase inhibitors, difficult to characterize because of spatial and temporal cellular events. It is, therefore, helpful for understanding cell pathways and facilitating drug discovery [1].

### Sample preparation

The sample preparation step is the key to successful phosphoproteomic-analysis. These include: (a) be snap-frozen; (b) include treatments with phosphatase inhibitors to avoid modifying phosphopeptides during sample work-up; and (c) avoid salts and detergents, which interfere with subsequent analyses. Using Immobilized metal ion affinity chromatography (IMAC), titanium dioxide metal-based chromatography (TiO_2_), zirconium dioxide (ZrO_2_) and sequential elution from IMAC (SIMAC), the negatively charged phosphopeptides are purified by their affinity to positively charged metal ions [1, 8].

During IMAC and TiO_2_ operations, simple and complex samples containing phosphopeptides and non-phosphorylated peptides are dissolved in an acidic solution to reduce the non-specific binding of acidic peptides, and to stimulate the electrostatic interactions between the negatively charged peptides, mainly phosphopeptides, and the metal ions. IMAC mainly elutes multiple-phosphopeptides while TiO_2_ chiefly elutes mono-phosphopeptides. Both resins have the drawback of binding acidic non-phosphorylated peptides (negatively charged peptides), as peptides containing acidic amino acid residues (*e.g.* glutamic acid and aspartic acid), can also bind to the metal ions. This drawback on IMAC (Fe^3+^) is circumvented *via* converting acidic amino acid residues to methyl esters and esterification of the acidic residues prior to the MS analysis. In addition, higher specificity is achieved and yield compared to IMAC (Fe^3+^) for the selective enrichment of phosphorylated peptides from model proteins when using 2,5-dihydroxybenzoic acid (DHB) with TiO_2_. In fact, more phosphopeptides are bound to the metal ions and more phosphopeptides can be isolated by using ammonium hydroxide as the eluent by use of glycolic acid in the loading buffer of TiO_2_. SIMAC is the combination of IMAC with TiO_2_ protocols. It includes improvements in both resins to be coupled in an efficient manner in order to allow enrichment of mono-and multiple-phosphorylated peptides in a single experiment. Mono-phosphorylated peptides mainly elute from IMAC (Fe^3+^) under acidic conditions whereas multi-phosphorylated peptides elute at high basic pH.

ZrO_2_ is another useful phosphopeptide enrichment prior to MS analysis and its principle is based on metal affinity chromatography such as IMAC and TiO_2_. The relevant clue related to ZrO_2_ is that it permits the isolation of single phosphorylated peptides in a more selective manner than TiO_2_ [12–24]. In addition, purification of phosphorylated proteins can be carried out *via* antibody-purification. This methodology is highly efficient when purifying tyrosine –phosphorylated proteins, and it can also be coupled to phosphoenrichments such as IMAC, TiO_2_, ZrO_2_ and SIMAC for further MS analysis. This is an important advantage as phosphorylation on tyrosines is under-represented by MS-assays, thus the use of specific antibodies to enrich tyrosine phosphorylated peptides from complex samples is of advantage [25].

When combining the previously mentioned phosphoenrichments with strong cation and anion exchange (SCX and SAX) or hydrophilic interaction chromatography (HILIC), large-scale phosphoproteomic studies of interest can be carried out successfully. During SAX operations, a negatively charged analyte is attracted to a positively charged solid support, and during SCX operations, a positively charged analyte is attracted to a negatively charged solid support. Both techniques were, for the first time, successfully coupled to IMAC, achieving greater recovery and identification by MS of important phosphorylated peptides originating from signalling pathways and membrane proteins respectively, therefore making it possible to carry out relevant scientific studies following those protocols described in the references previously detailed. In addition, today’s scientists use these tools to achieve important biological understanding. HILIC is a liquid/liquid extraction method among mobile and stationary phases (polar). On the surface of the stationary phase a water rich layer is obtained. Therefore, distribution of the analytes among these layers occurs. More polar compounds show stronger interaction with the stationary aqueous-layer than less polar compounds, taking place in stronger retention [26–28].

### Improvement in methodologies

Improvement in methodologies to enrich for phosphorylated residues from kinases is clearly necessary for clinical cancer research. This is difficult due to: (1) the low abundance of those signalling molecules within cells; (2) the stress/stimulation time-duration, of phosphorylated kinases; (3) the time adaptation over signalling pathways. To overpass this issue, several approaches-combinations can be applied: for example SIMAC coupled to SCX and LC-MSMS. In every case it is always necessary to optimise, by expertise proteomics, the right strategy (several proteomic combined tools) to achieve the clinical goal previously established. In addition, using ‘the combination of C_18_-TiO_2_-C_18_ chromatography, efficient phosphopeptide-loading conditions are increased for clinical samples containing kinases or low expressed proteins and more importantly, “*it is without compromise on sample loading or analysis based-times as, it permits the complementary measurement of the enriched phosphopeptides and their non-phosphorylated counterparts in following assays, making for quantitative phosphoproteomics”* [29]. In the combination of several phosphoproteomic strategies, signaling aberrant pathways involved in cancer progression can be understood, thus drug resistance can be reduced and therapies can be improved.

## Discussion

### Combining phosphoproteomics and bioinformatics for deciphering drug resistance and new therapies related to phosphorylation pathways in cancer

The proteomic community, EUPA and HUPO played very important roles, and today many hospitals are applying those proteomic advances to study the evolution of patients, as by combining proteomics to genomics data, many diseases which were not cured in the past, have now been improved [30, 31].

For example, D’Souza RC *et al.* [32] coupled assays of changes in protein expression, phosphorylation, protein interactions and transcriptional regulation, allowing a brilliant scheme of the dynamic signaling events underlying TGF-β-induced changes in cell behavior. Their study evokes that temporal regulation of different proteins could be a mechanism to arbitrate the effects of TGF-β in keratinocytes. They also showed that early TGF-β-signaling is a combination of pro- and anti-proliferative molecules. It is known that TGF-β signaling actively support cell-motility *via* the inducing of epithelial to mesenchymal transitions in cancer. Authors carried out time resolved assays of the phosphoproteomic profile of cultured human keratinocytes undergoing epithelial-to mesenchymal transitions and cell cycle arrest as a consequence of stimulation with TGF-β. They were able to quantify significant changes in around 3,000 phosphorylation sites regulated *via* TGF-β. Moreover, following their phosphoproteomic strategy, authors discovered that TGF-β induced phosphorylation of AKT and GSK3α. This study explains that the combination of system-level assays with knowledge of specific phosphorylated sites facilitate a deeper understanding of the mechanisms of crosstalk pathways.

Another example shows how phospho-quantitative assays in skin cancer (*via* SILAC mouse technology coupled to MS) can complement genetic assays, *via* different states using mouse models. Zanivan *et al.* [33] carried out phosphoproteomics to get the specific molecules involved in different cancer stages, observing that the cell growth and cell adhesion are altered pathways to driver malignant cells. Moreover authors observed that when coupling phosphoproteomics and prediction of kinase activity, in this research study, PAK4-PKC/SRC network was highly deregulated in SCC although it was not in papilloma. As they said “*This detailed molecular picture, both at the proteome and phosphoproteome level, will prove useful for the study of mechanisms of tumor progression*”.

The importance of the heat shock protein 90 (Hsp90) inhibitors as chemotherapeutic agents in diseases such as cancer is increasing, but their total consequences on the proteome are yet unknown. Sharma *et al.* [34] *via* quantitative-MS mapped map protein expression changes associated with the application of the Hsp90 inhibitor, 17-(dimethylaminoethylamino)-17-demethoxygeldanamycin (17-DMAG). In this study, they demonstrated that the activation of a heat shock response with induced expression of molecular chaperones, that re-fold misfolded proteins and proteases, can degrade irreversibly damaged polypeptides. Moreover, they were able to quantify 6,000 proteins in HeLa cells *via* SILAC. In spite of the high number of substrates related to Hsp90, *via* bioinformatics, they observed that the preferred ones were proteins related to DNA damage response, and protein kinases, especially tyrosine kinases. In addition, they observed that the inhibition of Hsp90 triggered to 34 % down- and 6 % up-regulation of the phosphoproteome. These assays illustrate the cellular response to Hsp90 inhibition at the proteome level and gives light on the mechanisms *via* Hsp90 which could be used to target cancer cells.

Rigbolt *et al.* [35] observed an interaction of DNMTs for early differentiation with the polymerase-associated factor 1- transcriptional elongation complex. It is able to link to DNMT target genes encoding OCT4 and NANOG. Therefore, this assay gives a possible molecular clue for silencing OCT4 and NANOG for differentiation cell step. The authors carried out quantitative proteomic and phosphoproteomic assays of human embryonic stem cells to observe cellular events for differentiation cells. They were able to identify around 7,000 proteins and 24,000 phosphorylation sites. From them, 50 % showed different expression-profiles for differentiation-step. Moreover, they got the phosphoproteome-core of human embryonic stem cells. The proteins which showed different phosphorylation-pattern are linked to several kinases, transcription factors and DNA methyltransferases. Thus, this study clearly shows a new molecular-connector for silencing the genes OCT4 and NANOG for differentiation-step.

### Phosphorylated peptides/proteins mass spectrometry data need to be validated

The incapacity to adequately validate MS/MS spectral assignments for each phosphorylated peptide is of importance in relation to the tendency towards massive phosphoproteomic data. If adequately carried out, the assignment of all abundant ions in the spectrum is required for spectral validation. This implies a large time commitment. In order to avoid this drawback, many mass spectrometry laboratories have used other search strategies and statistical methods to make an estimate of false positive identification rates. For phosphoproteomics, statistical validation is of great importance in comparison to the wider field of proteomics, given that each phosphorylation is defined, typically, by a single MS/MS spectrum. Using statistical methods, unfortunately it is not possible to know if any given phosphorylation site is correctly identified, as the MS/MS spectrum is not validated. For biologists with an interest in studying the function of these phosphorylation sites, these false positive identifications are particularly treacherous, due to the fact that the complete investigation of each phosphorylation site may take up to two years. As most biologists do not possess the expertise necessary to assess the accuracy of the assignment even if the raw MS/MS spectrum is provided, the situation worsens, and it is frequently wrongly assumed that all phosphorylation assignments published are correct. Manual validation of the phosphorylated peptides/proteins is, on the other hand, a good challenge, although it implies great expertise with “reading” the spectra [28–35]. In addition, it is essential to get the specific molecules activated-deactivated during cancer evolution of patients, as this implies relevant clues for unraveling good and bad prognoses related to drug resistance and new target-therapies. Phosphoproteomics is a powerful methodology which allows this issue, so that patients will be benefited.

### Validation of the phospho data

Here we would like to describe the manual validation of the phospho data (assignments of the phosphate group on specific amino acids) obtained in an MS experiment during CID (collision-induced dissociation) operations. When peptide ions are fragmented *via* CID, a series of y- and b- ions are formed. The peptide sequence is obtained by correlating the mass difference between peaks in the y-ion series or between peaks in the b-ion series with amino acid residue masses. The CID fragmentation mainly occurs on the peptide backbone, and sequence information is obtained. In relation to phosphotyrosine residues, partial neutral loss is observed (HPO_3_, 80 m/z) in MS2 mode, and the phosphate group on tyrosine (tyr) residues is more stable than on serine (ser) and threonine (thr) residues. In addition, the phospho-finger-print characteristic of phosphotyrosine is the phosphotyrosine immonium ion (~216 Da). *Via* MS3 mode, the ion originating from neutral loss (NL) of phosphoric acid (H_3_PO_4_) can be selected for further fragmentation. The selected ion is then automatically selected for further fragmentation after neutral loss fragmentation. Therefore, it is possible to add extra energy for the fragmentation of peptide backbone. Nevertheless, the MS3 mode requires that the phosphorylation on ser and thr residues are labile and conventional fragmentation *via* CID commonly results in the partial NL of H_3_PO_4_, (98 m/z) in MS2 mode. This is due to the gas phase β-elimination of the phosphor-ester bond and thus, dehydroalanine (ser ~69 Da) and dehydro-2-aminobutyric acid (thr ~83 Da) are generated [36–48].

More importantly, a library-database containing around 5,000 manually validated phosphopeptides product ions and related spectra, from around 330,000 CID fragment spectra analyzed in several LC-MSMS assays from new research studies was created and placed in Sequest (http://fields.scripps.edu/sequest/index.html) [49] and Mascot (http://www.matrixscience.com) [50–55]. Those CID spectra have been annotated and classified *via* Sequest and Mascot settings. Those databases apply the filter and search criteria described in their studies. In fact, with relation to the filtering, the resulting spectra data is manually validated *via* SILVER web application (http://llama.mshri.on.ca/cgi/SILVER/silver.cgi?id=665224) [56].

### Validation by bioinformatics softwares

Laboratories worldwide can now routinely carry out phosphoproteomic assays as owing to the fast improvement of both MS and efficient phosphopeptide-enrichments, scientists and clinicians have easy access to lists of thousands of phosphorylated peptides for further biological issues. The application of computational, statistical and predictive analytical methods is vital to answer the biologically relevant questions arising from these phosphor-data [57, 58].

It is also possible to carry out the validation more rapidly by bioinformatics software for proteomics assays useful for clinical research. For example, PhosphoSitePlus (http://www.phosphosite.org/homeAction.do) [58] consists of a database with a comprehensive collection of different PTMs including phosphorylation, ubiquitinylation, acetylation and methylation, and it was created by cell signaling technology. From all PTMs, 78 % are phosphorylation, 15 % ubiquitinylation, 6 % acetylation. An important feature of PhosphoSitePlus is its ability to generate high-throughput phosphoproteomics data, including both low-throughput and high-throughput experimental data, and rapid sharing of the newly generated data *via* its website.

On the other hand, The Human Protein Reference Database [59, 60] is one of the largest databases for the human proteome (http://www.hprd.org). It includes data from PTMs, sub-cellular localization of human protein, protein-protein interactions, enzyme-substrate relationships, tissue-expression disease-associations all which data is freely downloadable. Also, Phospho. ELM [61–63] (http://phospho.elm.eu.org) mainly contains the collection of manually curated phosphosites and information derived from small-scale experiments. It contains information on around 300 kinases, 8,000 substrates, and over 42,000 phosphosites [63].

### Examples of tools for validation potential analysis of the phospho data

Many computational approaches [64–73] are useful for phosphorylation networks studies of clinical cancer research, or for connecting kinases and phosphosites into a biological signaling network. Bioinformatics tools in this sense, for example, serve to design new drug-candidates from proteomic data. Below are some useful networks for reference.

The STRING-database (http://string-db.org), [64–66] was created to improve and increase the specificity properties from motif-based predictions. In this database there are over 20,000 site-specific interactions available *via* their website including nearly 4,000 phosphoproteins and 74 human kinases from 20 families and thus, interesting drug-resistances and toxicities studies can be carried out *via* STRING.

*Via* NetworKIN (http://www.networkin.info/index.shtml) [57, 68, 70] network context of kinases and phosphoproteins are incorporated *via* prediction algorithms. It contains sub-cellular compartmentalization, co-localization *via* anchoring proteins, scaffolds, temporal and cell-type specific co-expression. The algorithm applies neural-networks and position-specific score-matrices in order to assign proper phosphosites to kinases and related families. It consists of intrinsic preference of kinases for consensus of substrate motifs, thus, for example important true biomarkers can be validated coming from proteomics assays.

PANTHER (http://www.pantherdb.org) [71] is focused on inferring the phosphorylation of substrates by corresponding kinases, frequently referred as KSR. Performance of bioinformatics tools to predict binding substrate specificities of protein kinases originated from experimental identification of consensus sequence motifs recognized by the active sites of kinases. It is worth remembering that some phosphorylated residues by different kinases from the CDK and SRC families cannot be distinguished by their consensus sequences alone. Several of these general bioinformatics tools can be applied to capture the evidence and narrow them down to key players in the signaling network. In fact, the signaling network of TBK1- was deciphered as an emerging drug target *via* phosphoproteomics [64–74]. Authors applied several tools, such as gene ontology (GO) pathway enrichment analysis, motif analysis using the motif-x algorithm [75–77].

Finally, when obtaining data with mutated kinases, clinicians and scientists are interested in knowledge about which kinase-inhibitors can be applied for cancer treatments. Currently, a high-throughput kinase-inhibitor database is accessible, *via* K-MAP (http://tanlab.ucdenver.edu/kMap/kMapv1.0) [78] and can be used to complement and design future clinical assays to study resistances and toxicities.

There are a number of bioinformatics tools available for biomedical research (Ingenuity Pathway Analysis (www.ingenuity.com) [69], KEGG (http://www.kegg.jp) [70–72], GeneGo metaCore pathway analysis (www.genego.com) [73], iGPS for systematic analysis of protein phosphorylation networks from phosphoproteomics data; http://igps.biocuckoo.org/index.php) [75, 79], and PostMod for sequence based prediction of kinase-specific phosphorylation sites with indirect relationship (http://pbil.kaist.ac.kr/PostMod) [76] useful and available in specific reviews useful for clinical research. They are currently in use for cancer research and support proteomics to advance the human proteome sequence [77, 78, 80–90].

## Conclusion

With the advances in proteomics and in the near future the completed sequence of the human proteome, we envision we will have a better diagnoses and therapies for the diseases. Today, proteomics is applied in many international hospitals in order to obtain the information about the evolution of patients and obtain specific molecules/sights related to prognoses of various diseases, with benefit for more patients in the coming future.
